# Examining reach, dose, and fidelity of the “Girls on the Move” after-school physical activity club: a process evaluation

**DOI:** 10.1186/s12889-016-3329-x

**Published:** 2016-07-30

**Authors:** Lorraine B. Robbins, Jiying Ling, Ebru Kilicarslan Toruner, Kelly A. Bourne, Karin A. Pfeiffer

**Affiliations:** 1 College of Nursing, Michigan State University, 1355 Bogue Street, East Lansing, MI 48824 USA; 2 Health Sciences Faculty Nursing Department, Gazi University, Emniyet Mah. Muammer Yasar Bostanci Cad. No:16 06560, Besevler/Ankara, Turkey; 3 Department of Kinesiology, College of Education, Michigan State University, 27R IM Sports Circle, East Lansing, MI 48824 USA

**Keywords:** Randomized controlled trial, Intervention, Adolescents, Females, School

## Abstract

**Background:**

After-school programs represent a promising opportunity to assist adolescent girls’ in attaining adequate physical activity. Although evaluating the process of intervention implementation is important for determining if an intervention was delivered and received as intended, comprehensive information about process evaluation methods and results are rarely reported. The purpose of this article was to evaluate the reach, dose, and fidelity of a 90-minute after-school physical activity club offered 3 days a week. The club is 1 of 3 components included in a 17-week intervention designed for 5th-8th grade girls, the majority of whom were of minority and/or low socioeconomic status.

**Methods:**

A total of 24 schools (12 intervention; 12 control) and 56–67 girls per school (total *N* = 1519 girls) were included in the *Girls on the Move* group randomized controlled trial. At the beginning of each of 3 academic years (2012–2015), 8 schools per year were randomized to receive either the intervention (*n* = 4) or control condition (*n* = 4). To evaluate the club, data collected via surveys from girls, club coaches and managers, and process evaluators were analyzed. To evaluate the opportunity for physical activity provided by the coaches and managers, process evaluators used an observation tool based on the System for Observing Fitness Instruction Time and Academic Learning Time - Physical Education. Girls wore accelerometers every other week during the club time.

**Results:**

Mean attendance was 41 % with the average attendance in year 3 being higher than rates for years 1 or 2. Mean moderate-to-vigorous physical activity time was 21.85 minutes measured via accelerometry and 21.81 minutes observed by process evaluators. Satisfaction with the intervention was high. For the most part, process evaluators perceived the club was delivered as planned and reflected constructs of the Health Promotion Model and Self-Determination Theory. Areas contributing to success included using incentives and offering a variety of activities. Issues negatively impacting implementation included managing behavioral problems, having limited space for moderate-to-vigorous physical activity, dealing with inclement weather, and getting coaches to actively participate in all physical activities with the girls.

**Conclusions:**

This process evaluation provides important information to guide future school-based physical activity intervention delivery. Barriers to implementation have been identified. Ways to overcome them warrant consideration when designing physical activity interventions. Research is needed to test innovative approaches for enhancing attendance and increasing girls’ moderate-to-vigorous physical activity in after-school programs.

**Trial registration:**

ClinicalTrials.gov Identifier NCT01503333. Registered 23 December, 2011.

## Background

Regular physical activity (PA) promotes health and reduces the risk of physical and psychological chronic conditions, such as cardiovascular disease, obesity, and depression [1, 2]. Although participating in moderate-to-vigorous PA (MVPA) at least 1 h per day is recommended for children and adolescents [3], only a low percentage of these populations actually achieves the U.S. Department of Health and Human Services recommendation [4]. Even more disconcerting is that participation in MVPA declines significantly as age increases, particularly among girls during adolescence [5, 6]. Specifically, among 6–11-year-old boys and girls, 48.9 and 34.7 %, respectively, attain the recommended level of PA; while among 12–15-year-old boys and girls, only 11.9 and 3.4 %, respectively, meet the recommendation [4]. According to the 2013 U.S. Youth Risk Behavior Survey, slightly over a quarter (27.1 %) of high school adolescents meet the recommendation, with boys achieving the recommendation more than girls, 36.6 and 17.7 %, respectively [7]. To assist adolescents in increasing their PA, school-based interventions, such as PA clubs, physical education classes, and other PA programs, are needed, especially for girls [8, 9].

Schools are in an ideal position to promote PA because required school attendance offers potential opportunities before, during, and after school to positively change the behavior [10]. Capitalizing on this situation, several researchers have focused on evaluating the effectiveness of school-based interventions in increasing PA [11–14]. Despite a concerted effort, 2 systemic reviews showed that school-based PA interventions had either little or no effect on MVPA [10, 15], even though in one of the reviews, the duration, frequency, and intensity of the interventions and combination or type of PA offered to children and adolescents, aged 6 to 18, varied greatly across the included studies [15].

Similar results were noted in both a systematic review and a meta-analysis focusing on PA interventions conducted with girls. In the systematic review, Camacho-Miñano and colleagues noted that only 7 of the 21 included studies had both a high methodological quality and favorable effect on PA [16]. Unfortunately, of the 7 studies, 6 relied on a self-report measure of PA [16]. One of the 7 studies included accelerometers with a modest effect of 1.6 min per day of MVPA that occurred only in its final year [17]. In the meta-analysis that included 45 studies, Pearson and colleagues reported a significant, but small, average intervention effect of .35 on PA [18]. In addition, the effect size of .29 for 6 interventions using an objective measure of PA was smaller than the value of .38 for 26 interventions involving self-report. Of concern was that self-reported PA among adolescents, which often shows weak validity due to recall bias and error, may have inflated the effect [18]. Regardless, both Camacho-Miñano et al. and Pearson et al. concluded that increasing girls’ PA is a complex task and multi-component interventions are most effective for meeting the challenge [16, 18].

The findings from these previously conducted studies not only indicate that effective interventions have yet to be identified, but also underscore the need to thoroughly and critically examine the delivery of each component of any multi-component intervention [19]. The preferred method for obtaining detailed evaluative information about the delivery of an intervention in order to interpret its potential effects is known as process evaluation [20]. A comprehensive understanding of reasons underlying differences between expected and observed outcomes can lead to improvements in the design, effectiveness, and efficiency of intervention delivery [21].

Process evaluation generally involves the measurement of intervention reach, dose, and fidelity [22, 23]. Assessment of “reach” provides evidence on whether and how the intended audience participates in the intervention or specific intervention components [24]. “Reach” is usually reported as the proportion of participants who attend sessions or have exposure to various program elements. Evaluation of the “dose” is aimed to capture the quantity of intervention provided by examining what was received by and delivered to the participants, and can be evaluated by dose delivered and dose received (exposure and satisfaction). *Dose delivered*, which reflects implementation completeness or the efforts and behaviors of the interventionists to provide the opportunity or planned amount of intended units of the intervention, can be measured by direct observation utilizing a tool designed specifically for this purpose [25]. *Dose received* (exposure) refers to the extent to which participants are actively engaged with or receptive to the intervention and resources provided [22]; and *dose received* (satisfaction) includes participants’ satisfaction with the program, as well as staff and/or investigators [22]. Therefore, to determine participants’ active engagement in PA, *dose received* (exposure) can be evaluated using objective measures, such as heart rate monitors, accelerometers, pedometers, or any other PA trackers, such as Fitbits. *Dose received* (satisfaction) can be measured via survey. Measurement of “fidelity”, which can also be accomplished via survey, assists in determining the extent to which the intervention is consistent with the conceptual framework on which it is based [22]. Information acquired from the 3 dimensions, including dose, reach, and fidelity, can prevent a Type III error, an erroneous conclusion that the intervention itself was not effective because of its own inherent inadequacy [26].

The purpose of this study was to evaluate the reach, dose and fidelity of an after-school PA club, 1 of 3 components included in a 17-week intervention called *Girls on the Move* [27]. In order to provide a comprehensive evaluation of the PA club and maintain an article of reasonable length, evaluative findings related to the following other 2 intervention components were not included: 1) 2 face-to-face motivational interviewing sessions with a counselor scheduled to be conducted during the school day (1 at the beginning; the other at the end of intervention period), and 2) an interactive Internet-based session in which each girl received individually tailored and motivational feedback messages gleaned from her responses to survey items administered via an iPad during the school day (midpoint of the intervention period). Few studies included a robust process evaluation of a PA intervention for girls [23, 28–32]. Although evaluative findings on the motivational interviewing and Internet-based sessions were not included in this article, which may be identified as a limitation, the information presented from this study is expected to make an important contribution to the limited body of knowledge that currently exists on implementation of an after-school PA program for girls.

## Methods

### Participants

**Fig. 1:**
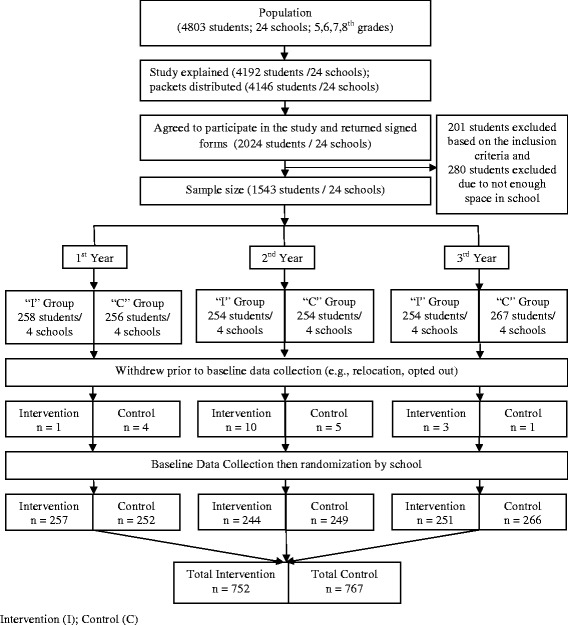
Origin and flow of participants in the study (*N* = 1519)

A total of 24 racially diverse urban (inner-city) schools (12 intervention; 12 control) in the Midwestern U.S. were included in the *Girls on the Move* group randomized controlled trial. At the beginning of each of 3 academic years (2012–2015), 8 schools per year were randomized to receive either the intervention (*n* = 4) or control condition (*n* = 4). The sample ranged from 56–67 girls per school (total *N* = 1519 girls). To be included in the study, girls had to meet the following criteria: 1) be enrolled in 5th-8th grade; 2) have written parental/guardian consent to participate in the entire study, including the data collection; 3) be available for follow-up data collection 9 months after the intervention ends; 4) agree to school random assignment to intervention or control conditions; and 5) be able to read, understand, and speak English. Exclusion criteria were: 1) involved in or planning to be involved in PAs that involve MVPA and require participation 3 or more days per week after school; and 2) a health condition that would prevent engagement in MVPA. Figure 1 depicts the origin and flow of participants in the study. Detailed information on the study’s method can be found in the published study protocol [27]. This article focuses only on the intervention group (58–66 girls per intervention school).

### Ethics approval and consent to participate

The study was approved by the Michigan State University Institutional Review Board. School administrators provided permission to conduct the study in their respective school districts. All participating students and their parents/guardians signed assent and consent forms, respectively.

### Design

A group randomized controlled trial was conducted. In the fall of each of the 3 academic years of the intervention, 8 schools were paired based on the following criteria: 1) school type (e.g., academic grades offered), 2) school size, 3) racial proportion (white vs. non-white race), and 4) percentage of students receiving the free and reduced-price lunch, an indicator of low socioeconomic status (SES). After baseline data collection, the paired schools were randomized to intervention or control conditions to decrease bias. Each year, the measurement and intervention teams functioned independently with no interaction so as to blind members of the former group to each school’s randomization status.

### Intervention

Based on the integration of the Health Promotion Model (HPM) [33] and Self-Determination Theory (SDT) [34], the intervention was designed to facilitate long-term attainment of adequate MVPA by enhancing girls’ perceptions of perceived benefits (HPM), self-efficacy (HPM), enjoyment (HPM), social support (HPM), role models (HPM), autonomy (SDT), relatedness (SDT), and competence (SDT) and reducing barriers relative to PA (HPM) [33, 34].

The PA club was offered to girls 3 days/week from Tuesday through Thursday for 17 weeks after school with the exception of school breaks (holidays and half days) or school cancellations due to inclement weather. The 90-min PA club was designed to include organizational tasks (e.g., recording attendance) and a healthy snack with a bottle of water before activities (10 min), warm-up activities (5 min), MVPA (60 min), cool-down activities (5 min), and organizational tasks (e.g., putting equipment away) and healthy snack after activities (10 min). Healthy snacks included fruits, vegetables, low-fat yogurt, or cheese. A major objective was to engage the girls in MVPA for at least 50 % of the allotted 60-min period, similar to what is recommended for physical education classes [35–37].

For each intervention school, the intervention coordinator hired a PA club manager and 3–4 PA club instructors, all of whom had to have recent prior experience conducting community- or school-based PA programs that involved a similar age group of girls. To create a situation comparable and equal in importance to what occurs in girls’ sports, the girls agreed to refer to each instructor as “Coach (first name of instructor).” To avoid lack of transportation as a barrier [38], buses were provided to take the girls home after every club session.

### Training

Prior to the start of the intervention each year, all PA club coaches and managers completed 8 h of face-to-face didactic and interactive training led by the intervention coordinator. At the beginning of the training, each coach and manager received a 313-page manual, including policies/procedures, PA modules on various sports skills and fun physical education games, and the curriculum format to be implemented in the club. The manual was created by the intervention coordinator in consultation with an exercise physiologist and curriculum development expert, all of whom had extensive expertise in designing and/or conducting PA programs for children and adolescents. The didactic portion of the training included a comprehensive review of the manual contents to ensure that coaches and managers understood all expectations regarding club delivery. The interactive part involved first the intervention coordinator and then small groups of coaches and managers leading a PA. Constructive feedback was provided.

The intervention coordinator trained all coaches and managers to offer a motivating environment with “fun” PAs (enjoyment) to help girls perceive the benefits of PA and improve their PA skills (competence and self-efficacy) with social support and role modeling from both peers and coaches (relatedness). The intervention coordinator emphasized the need for all PA club coaches to actively participate in the PAs with the girls during every club session. Observing girls engaging in PAs by themselves was discouraged. The intervention coordinator also instructed the coaches and manager to: 1) offer 2 different PAs (or even 3 if deemed necessary) each day so girls could choose the PA that they wanted to engage in (autonomy) and 2) encourage girls to continue their PA outside the club and strive toward attaining the U.S. Department of Health and Human Services PA recommendations. Coaches were asked to give clear messages before each PA to inform girls in advance about what they can expect during the PA session. The managers and coaches were also informed that a process evaluator would be visiting some sessions periodically to observe the club activities so that information could be obtained to assist the intervention coordinator in identifying ways to help them strengthen club delivery.

The intervention coordinator also met with the PA club coaches and managers at each school for at least 1 h every month throughout the intervention period to discuss any issues and reinforce policies/procedures. In every monthly meeting with all managers and coaches at each school, the coordinator reiterated to all coaches that their active PA participation with the girls was expected. The intervention coordinator also consistently provided feedback to all coaches and managers on the latest process evaluation results to share positive findings and stimulate discussion if needed on ways to improve intervention delivery.

### Procedure

The PA club was evaluated via attendance records, accelerometers, and session observations. Table 1 presents the process evaluation methods for the PA club. Data were collected by the intervention coordinator, PA club coaches and managers, and independent process evaluators. Accelerometer data were analyzed by research assistants trained by the study’s measurement coordinator. The blinded process evaluators and research assistants analyzing the PA data were not informed about the intervention or study outcomes.

**Table 1:** Process evaluation methods for the physical activity club

Characteristic	Data sources	Instruments/measurement	Timing	Data collectors or survey completers
Reach	• Attendance records of girls	• Attendance sheets & iPad	• Daily	• PA club manager
Dose received (exposure)	• ActiGraph GT3X-plus counts	• 5 girls randomly selected to wear accelerometer	• Every other week	• PA club coaches and manager
Dose received (satisfaction)	• Process evaluator observing club • Girls’ evaluation • PA club coaches’/managers’ evaluation	• 2-item survey to evaluate receptivity of intervention by process evaluators • 2-item survey to evaluate receptivity of intervention by girls • 3-item survey to evaluate receptivity of intervention by girls and the overall club as perceived by club managers and coaches	• Weeks 3, 9, & 15 • Post-intervention • Post-intervention	• Process evaluator • Girls • PA club coaches and managers
Dose delivered	• PA club observations	• Combination of SOFIT and ALT-PE instruments; a stopwatch to record and ruler to mark beginning and end of activity times in minutes were used.	• Weeks 3, 9, & 15	• Process evaluator
Fidelity	• Survey	• 8-item survey to evaluate extent to which intervention reflects conceptual framework • 11-item survey completed by girls to assist in determining extent to which intervention reflects conceptual framework • 11-item survey completed by club managers and coaches to assist in determining extent to which intervention reflects conceptual framework	• Weeks 3, 9, & 15 • Post-intervention • Post-intervention	• Process evaluator • Girls • PA club coaches and managers

*ALT-PE* academic learning time - physical education, *PA* physical activity, *SOFIT* system for observing fitness instruction time

During each PA club day at every intervention school, 3 coaches were scheduled to deliver the PA sessions, and a club manager tracked attendance, prepared the list of girls who needed bus transportation, and managed behavioral issues. The coaches in the 12 intervention schools ranged in age from 20–50 years, and all were female except 3. At the end of every club day, each PA club manager or team reported directly to the intervention coordinator, who was either housed at the university or present at the club, so that the intervention coordinator could record the types of activities offered each day in the PA club, provide support to the manager and PA club coaches as needed, and provide necessary equipment or assist with maintaining proper functioning of the equipment.

To create a manageable group size, ensure adequate supervision to keep disruptive behavior down to a minimum, and provide opportunities for the girls to have some choice regarding their PA, girls were divided into 1, 2, or 3 groups (number of groups varied each day depending on club attendance) either randomly or stratified by grade level. The number of girls in each group ranged from 7–27, and 1 or 2 coaches led each group. Although the number of coaches assigned to a school’s club varied at times based on the usual number of girls attending the club, the intervention coordinator strove to ensure a ratio of approximately 15 adolescents to 1 coach, as recommended in previous similar studies [39]. On rare occasions (e.g., coach absent or ill), a coach managed more than 15 girls.

Two to 3 types of MVPA were always offered every day at each club, but the girls were not always divided into 2 or 3 groups unless attendance was high (>15 or >30 girls total present, respectively). Each group rotated either once after completing an initial 30-min MVPA session, or sometimes twice, if the first MVPA session lasted 20 min, to a different MVPA session until participation totaled 60 min. Most, or all, girls in each group participated in 2 types of activities for 30 min each during the majority of the 60-min MVPA sessions offered in each school. At times, some girls chose to remain in the same MVPA session longer than 20 or 30 min, and coaches allowed them to do so. The various types of MVPA offered during the 20- to 30-min sessions included: 1) fun games (tag; flicker ball; kickball; boot camp stations, including sit-ups, squats, mountain climbers, agility exercises with a ladder; use of hand weights, hula hoops, and jump ropes; activities with Omnikin ball; scavenger hunts; capture the flag; cup stacking; fitness challenge); 2) dance (video games projected on a large screen, zumba taught by a coach having expertise in teaching zumba, line dances popular among girls, dance fitness routines and aerobics; pilates); and 3) walking or sport skills (soccer, basketball, volleyball, lacrosse, running, weight lifting, tennis, martial arts, track, floor hockey, badminton; ultimate disc). Small-sized groups and rotations to coaches offering a different type of MVPA provided girls with the opportunity to engage in and develop skills in different activities and avoid boredom. The approach also prevented cliques from forming [39]. If only a small number of girls attended the PA club (≤15 girls total), they remained as a single group and participated together in the 2 types of MVPA offered for the day.

In year 1, at the beginning of every club day, coaches selected 2 or 3 PAs (based on the number of girls attending) from an ‘activity box’ and then determined whether the majority of girls were interested in engaging in the selected PAs by allowing girls to vote. This approach provided too much freedom to choose, and achieving consensus among the girls was a challenge. Coaches expressed concern about not being able to plan in advance and requested increased structure regarding the PAs offered. In year 2, a ‘horizontal calendar’ approach was used with the same 2 or 3 PA modules being offered for 1 or 2 weeks. So, for example, if a PA module focused on basketball, then girls would learn at least 1 new basketball skill per day during the 1- or 2-week period (e.g., day 1, dribbling; day 2, shooting; and so on). The issue with this approach was that girls did not come to the club on all 3 days per week if they did not like the PA offered during a particular week or 2. In year 3, a ‘vertical calendar’ approach was implemented with the 2 or 3 PAs offered daily varying from day to day in a single week, but remaining the same on a particular weekday for 3 weeks. Positive verbal feedback from the managers, coaches, and girls indicated that the latter approach was well-received. The approach was also helpful in hiring the most qualified coaches, some of whom could only conduct the club sessions on the same day each week.

Also, in year 3, at the beginning of the first club day each week, the manager and coaches asked for a few girls to share what PAs they had done by themselves or with others during the past 4 days when the club was not being conducted. At the beginning of the middle club day each week, the manager and coaches asked for a girl to share a strategy/tip that she used or could use to attain the recommended 60 min of MVPA daily (e.g., dance to music at home). At the beginning of the last club day each week, the manager and coaches shared and also asked for a few girls to share what PAs they had planned to do by themselves or with others over the next 4 days when the club was not conducted so they could continue to try to attain adequate MVPA.

A point-system was created to manage negative behavioral issues identified as being a common occurrence in several after-school programs [28, 40]. Girls who attained 4 points per day (1 point each for attending, arriving on time, actually participating in the activities offered, and exhibiting appropriate behavior based on a mutually agreed-upon code of conduct established by the coaches, managers, and girls at the initial club session) had an opportunity to receive an incentive.

Incentives and related strategies varied across the intervention years. In year 1, $25 raffles were held at the end of each club week. Every day that a girl attended the club and received the full number of points, she received a ticket to enter into the week’s raffle. If a girl did not win the raffle drawing in a particular week, she received a small consolation prize (e.g., lip gloss rings, sunglasses, notebooks). Although girls were able to choose their prize from a broad selection of items, they expressed some dissatisfaction with the “small” prizes and the fact that sometimes girls who did not attend regularly won the raffle. Based on the year 1 feedback and recommendations from the girls, we allowed girls participating in the intervention in year 2 to accumulate points to exchange for a “large” item (e.g., athletic socks, movie passes, water bottles, basketball, iPod shuffle) from a *Girls on the Move* “gift” store. However, the girls did not want to wait to accumulate points to exchange for a store item. Therefore, in year 3, an immediate gratification monetary incentive was used. At the end of each week, girls received $5.00 in cash for attaining the full number of points on all 3 days, $2.00 for attaining the full number of points on 2 days, and $1.00 for doing so on 1 of the 3 possible attendance days. This latter approach increased club attendance from 38 % in year 1 and 37 % in year 2 to 49 % in year 3. The incentives were essential for not only increasing girls’ attendance but also reducing behavioral problems interfering with the delivery of the club activities by the coaches.

Team members, including the intervention coordinator and club managers, worked together to contact parents/guardians of girls who missed a full week of the PA club sessions over the initial 3-weeks of the intervention. To ensure consistency across the telephone conversations, all team members used a script developed specifically for this study as a guide to: 1) provide positive communication strategies to parents/guardians to help them gently encourage their daughters to attend the club; 2) emphasize reasons for and importance of having their daughters attend; 3) explore barriers that prevent their daughters from attending the club and strategies to overcome them; 4) review national PA and screen time recommendations for youth [3, 41]; and 5) discuss ways that parents/guardians could help their daughters attain adequate PA outside the PA club so that their daughters could meet the recommendations calling for daily PA [3, 41].

### Process evaluation

#### Reach

Reach was assessed by club attendance rates. Each day, the PA club manager asked the girls to sign attendance sheets. The PA club manager then counted the number of girls present and compared the number to the signatures on the attendance sheets. The PA club manager entered the attendance data into an Internet-based program created for this study and delivered via an iPad.

#### Dose

To determine *dose delivered* across all 3 years of the intervention, a total of 7 independent process evaluators, each holding either a Bachelor’s or Master’s Degree in Kinesiology or completing the final year of their Bachelor’s Degree in Kinesiology program, performed PA club observations to evaluate the opportunity for MVPA provided by the coaches/managers to the girls. The researchers instructed the process evaluator to try to be as inconspicuous as possible when observing the sessions (e.g., sit quietly in a section of the activity room, such as a distant corner, and avoid sharing any comments about the session with the managers/coaches). Because most club days at each school included 2 or 3 small groups of girls, the process evaluator followed and observed only the group of girls that included those wearing the accelerometers at the time of the evaluation. This procedure was important to evaluate the extent to which girls were physically active when provided the opportunity and to examine the consistency between the recorded accelerometer data and minutes of PA observed and reported by the process evaluators. All girls in the group were observed. Process evaluations across all 3 years were conducted on weeks 3 (early), 9 (midpoint) and 15 (near the end) of the intervention [42]. The process evaluators used a stopwatch to record activity times in minutes, and completed an observation form including a ruler in minute increments to mark the time duration for snack/check-in, light PA, seated/standing activity, management, instruction, MVPA, and snack/check-out. The observation form was developed for this study by combining relevant items of the following 2 instruments: the System for Observing Fitness Instruction Time (SOFIT) [43] and Academic Learning Time - Physical Education (ALT-PE) [44]. The study’s measurement coordinator trained the process evaluators to perform the club observations and collect the evaluative data.

Additionally, club coaches and managers responded online via Survey Monkey to 3 items: 1) Training sessions that were conducted prepared me for my coach/manager duties; 2) Supplies necessary to perform my duties were provided by the university staff; and 3) In my opinion, the design/structure of the after-school club is appropriate for girls this age. Response choices were: 1 = disagree a lot, 2 = disagree a little, 3 = agree a little, and 4 = agree a lot.

*Dose received (exposure)* was evaluated by girls’ PA level during the club. The measurement coordinator trained the PA club managers to randomly select (i.e., choose every 5^th^ girl) 5 girls per school every other week to wear the ActiGraph GT3X-plus, a lightweight accelerometer that has been shown to be reliable and valid for assessing MVPA [45, 46]. The ActiGraph records acceleration counts from which minutes of MVPA and number of steps are estimated [46]. Count thresholds were used to determine PA intensities: moderate-intensity: 574–1002 counts/15 s, and vigorous-intensity: >1002 counts/15 s [47, 48].

To determine *dose received (satisfaction),* girls completed a 2-item self-report survey at the end of the intervention to report their satisfaction with the activities offered and club managers/coaches. Items were: 1) I liked the physical activities we did, and 2) I liked the coaches. During each club visit, process evaluators used a 2-item survey to evaluate if girls appeared to like the PAs conducted and their coaches by responding to the following items: 1) The girls appeared to like the physical activities conducted, and 2) The girls appeared to like their coaches. At the end of the intervention, club managers and coaches responded via Survey Monkey to 2 items for assessing their perceptions regarding receptivity of the intervention by girls: 1) The girls like the activities I conducted in the PA club, and 2) The girls like me as a coach in the club. They also responded to an item about the overall after-school club: In my opinion, the design/structure of the after-school club is appropriate for girls this age. Response choices for all items ranged from 1 = disagree a lot to 4 = agree a lot.

#### Fidelity

An 8-item survey including a Likert scale, which was adapted from one employed in prior pilot work [49], was used by the process evaluators to evaluate the fidelity or the extent to which the intervention, as delivered by the coaches, reflected the conceptual framework. To further determine the extent to which intervention reflected the conceptual framework, at post-intervention, girls completed an 11-item survey, and the managers and coaches responded to a different 11-item survey (see Table 2 for specific survey items and response choices).

**Table 2:** Survey items completed by girls, coaches/managers, and process evaluators

Group	Survey items
Girls	The coaches gave me some choice in selecting the physical activity I wanted to do.
	The club was fun.
	The club helped me increase my physical activity.
	The club coaches helped me see a lot of reasons for doing physical activity.
	The coaches helped me solve problems that stop me from being active.
	The coaches helped me see that I can be active.
	The coaches made me want to get more physical activity in the after- school club.
	The coaches made me want to get more physical activity outside the club.
	I felt connected to the girls in the club.
	I was able to relate to the coaches.
	The club helped me improve my activity or sports skills.
Coaches/managers	I gave the girls some choices in selecting the physical activities in the club.
	I made the club fun for the girls.
	I helped girls increase their moderate to vigorous physical activity.
	I helped each girl see a lot of reasons for doing physical activity.
	I helped each girl rise above problems that stop her from exercising, being active, or doing sports.
	I helped increase each girl’s confidence for doing physical activity.
	I motivated each girl to increase her moderate to vigorous physical activity in the club.
	I motivated each girl to get regular moderate to vigorous physical activity outside the club.
	I helped each girl feel connected to me and other girls in the club (so she felt a sense of belonging in the group).
	I helped each girl increase her skills for doing physical activity or sports.
	I was a good role model for physical activity.
Process evaluators^a^	Gave girls some choice (e.g., re: starting station; other).
	Used positive praise to reinforce good performance/behavior.
	Appeared to be prepared to lead the session.
	Emphasized the need to be physically active outside the club.
	Made the club fun for the girls.
	Motivated each girl to increase her moderate to vigorous physical activity in the physical activity club.
	Helped each girl feel connected to others in the club (so she felt a sense of belonging to the group).
	Helped each girl increase her skills for doing physical activity or sports.

*Note*. Process evaluators’ 8-item scale response choices included: 1 (disagree a lot), 2 (disagree a little), 3 (agree a little), and 4 (agree a lot). Eleven-item surveys completed by girls, managers, and coaches had response choices similar to those used by process evaluators, except for the addition of the following 5th response choice to the survey for the coaches and managers: Does not apply to me

^a^Process evaluators evaluated coaches/managers

### Analysis

Data obtained in the study were analyzed with the Statistical Package for the Social Sciences (SPSS) 22 package, and the frequency values and percentage distributions were presented. The variables’ compliance with the normal distribution was examined with visual (histogram and probability graphics) and analytical (Shapiro-Wilk Test) methods. The Chi-square test or Fisher’s test (in the cases where the values observed in the cells did not meet the assumptions of Chi-square test) was employed to compare the nominal data among intervention years. The Kruskal-Wallis (not normally distributed) or one-way analysis of variance (ANOVA; normally distributed) test was conducted to compare the ordinal data among intervention years. If significant, the Mann–Whitney or Tukey post-hoc test was used. While investigating the association between observed MVPA and accelerometer-measured MVPA, the Pearson product–moment correlation test was applied. Absolute correlation values from 0.81 to 1.00 were considered as very strong, .51 to .80 as strong, .31 to .50 as moderate, and .00 to .30 as weak [50]. The significance level was set at 0.05.

## Results

### Demographic characteristics

A total of 752 5^th^-8^th^ grade girls participated in the intervention. The average age for girls was about 12 years. Nearly half (45.1 %, *n* = 339) were African Americans, and 14.8 % (*n* = 111) were Hispanic. Slightly over 3/4 (77.6 %, *n* = 576) of the girls were eligible to receive the free or reduced-price lunch at school. Table 3 displays the baseline demographic characteristics of the participants in the intervention group.

**Table 3:** Baseline demographic characteristics of the intervention group (*N* = 752)

Demographic Variables	Year 1	Year 2	Year 3	Total
Age				
Mean ± SD	11.74 ± 0.75	12.68 ± 0.91	11.76 ± 1.00	12.05 ± 0.99
Min.-Max.	(10–14)	(11–15)	(10–15)	(10–15)
Grade *n* (%)				
5th	34 (13.2 %)	-	73 (29.1 %)	107 (14.2 %)
6th	146 (56.8 %)	59 (24.2 %)	81 (32.3 %)	286 (38.0 %)
7th	77 (30.0 %)	128 (52.4 %)	97 (38.6 %)	302 (40.2 %)
8th	-	57 (23.4 %)	-	57 (7.6 %)
Race *n* (%)				
African American	142 (55.3 %)	99 (40.6 %)	98 (39.0 %)	339 (45.1 %)
White	64 (24.9 %)	84 (34.4 %)	66 (26.3 %)	214 (28.4 %)
Mixed and other races	51 (19.8 %)	61 (25.0 %)	87 (34.7 %)	199 (26.5 %)
Ethnicity *n* (%)				
Hispanic or Latino	28 (10.9 %)	32 (13.1 %)	51 (20.3 %)	111 (14.8 %)
Not Hispanic or Latino	215 (83.7 %)	198 (81.2 %)	189 (75.3 %)	602 (80.0 %)
Missing	14 (5.4 %)	14 (5.7 %)	11 (4.4 %)	39 (5.2 %)
Free/Reduced Lunch *n* (%)				
Yes	188 (73.2 %)	196 (80.3 %)	192 (76.5 %)	576 (76.6 %)
No	51 (19.8 %)	24 (9.8 %)	38 (15.1 %)	113 (15.0 %)
Missing	18 (7.0 %)	24 (9.9 %)	21 (8.4 %)	63 (8.4 %)

*SD* Standard deviation

### Reach

The mean number of PA club days offered was 49.55 ± 1.94 over the 17-week period. Although we had planned for 51 club days (3 days × 17 weeks), the number of days that the club was offered in each school varied slightly for the following reasons: no school on a certain day, half-day sessions, cancellations resulting from severe winter weather, and parent-teacher conferences requiring additional school space. Across the 3 years, the total mean attendance at the PA club was 20.54 ± 16.50 days, equivalent to 41 % attendance (Table 4). The average attendance in year 3 was higher than rates for year 1 or 2 (49 % vs. 38 %, *p* = .002; 49 % vs. 37 %, *p* < .001; F_(2,750)_ = 8.88, *p* < .001). Overall, the percent of eligible girls attending continuously decreased over time from 65.9 % at the beginning of the club to 37.9 % at its end.

**Table 4:** Reach: physical activity club attendance

	Year 1	Year 2	Year 3	Total
Club Days offered				
Mean ± SD	51.51 ± 1.13	47.72 ± 0.81	49.31 ± 1.46	49.55 ± 1.94
Median (Min-Max.)	52.0 (50–53)	47.0 (47–49)	50.0 (47–51)	50 (47–53)
Club Days attended				
Mean ± SD	19.80 ± 16.72	17.68 ± 15.91	24.08 ± 16.27	20.54 ± 16.50
Median (Min-Max.)	18.0 (0–51)	13.0 (0–47)	25 (0–51)	19.0 (0–51)
% attendance				
Mean ± SD	0.38 ± 0.32	0.37 ± 0.34	0.49 ± 0.33	0.41 ± 0.33
Median (Min-Max.)	0.35 (0–0.98)	0.28 (0–1)	0.51 (0–1)	0.38 (0–1)

### Dose

To determine *dose delivered*, a total of 93 evaluations were conducted across the 3-year period: year 1 = 22 (the goal was 24 evaluations; 1 was cancelled due to time conflict, and 1 was lost because a research assistant misplaced the completed evaluation form); year 2 = 35 (the goal was 36 evaluations; one was cancelled due to inclement winter); and year 3 = 36. As shown in Table 5, the mean observed PA club MVPA time for the 3-year intervention was 21.81 ± 12.69 min. Results from the post-hoc tests showed that: 1) compared to year 1, light PA and seated/standing time were higher during years 2 and 3; 2) in year 2, management time was lowest, but overall program time was highest; and 3) snack check-in and check-out time was highest, but observed MVPA time was lowest in year 3.

**Table 5:** Dose delivered and received: process evaluator observation and accelerometers

	Year 1 (*n* = 22)	Year 2 (*n* = 35)	Year 3 (*n* = 36)	Total (*n* = 93)	Test statistic	*p*
Dose delivered: observed time	Mean ± SD	Mean ± SD	Mean ± SD	Mean ± SD		
Snack-check in	9.19 ± 4.30	11.06 ± 3.67	14.05 ± 3.93	11.77 ± 4.35	19.72^b^	<.001*
Light PA	6.10 ± 9.05	11.73 ± 9.95	10.52 ± 8.38	9.93 ± 9.32	9.61^b^	.008*
Seated/standing activity	3.37 ± 5.21	12.08 ± 9.02	8.70 ± 6.88	8.71 ± 8.09	19.16^b^	<.001*
Management	24.73 ± 7.34	18.08 ± 8.07	23.15 ± 8.33	21.62 ± 8.41	5.73^a^	.005*
Instruction	6.51 ± 5.07	5.75 ± 4.56	6.69 ± 4.04	6.29 ± 4.47	1.33^b^	.516
Observed opportunity for MVPA	24.51 ± 9.13	27.53 ± 14.82	15.15 ± 8.25	21.81 ± 12.69	11.25^a^	<.001*
Snack/check out	6.27 ± 3.57	6.25 ± 3.93	11.68 ± 4.03	8.36 ± 4.67	34.01^b^	<.001*
Overall program	88.57 ± 5.77	92.47 ± 3.57	89.94 ± 6.19	90.57 ± 5.42	7.92^b^	.019*
Dose received: accelerometer-measured time						
MVPA	23.43 ± 7.12	19.66 ± 5.60	20.39 ± 5.74	21.85 ± 6.16	2.76^a^	.069
Step counts	2975 ± 764	2820 ± 888	2749 ± 793	2826 ± 820	.49^a^	.617

*Significant at *p* < .05

^a^One way ANOVA

^b^Kruskal Wallis test

To evaluate *dose received (exposure)*, the mean accelerometer-measured MVPA time was 21.85 ± 6.16 min, and the average number of steps was 2826 ± 820. The accelerometer-measured MVPA was significantly related to the observed MVPA (*r* = .42, *p* < .001). No differences were found according to intervention years. Details are presented in Table 5.

To estimate *dose received (satisfaction)*, 88 of the 93 (95.7 %) observations by the process evaluators indicated that the girls liked the PAs conducted in the club, and all agreed that girls liked their club instructors. A total of 451 girls completed the satisfaction survey after the 17-week intervention. On average, 87.8 % (*n* = 396) liked the activities offered in the club, and 85.4 % (*n* = 385) liked the club coaches/managers. Girls’ perceived satisfaction on activities (year 1: 80.8 %, year 2: 90.1 %, year 3: 93.5 %), and coaches/managers (year 1: 79.6 %, year 2: 83.2 %, year 3: 93.5 %) increased significantly over the years (*p* ≤ .001). Girls’ perceived satisfaction with activities positively influenced their club attendance (F = 8.85, *p* < .001), with girls liking the activities a lot having the highest attendance (54 %), as compared to girls liking the activities a little (40 %) and those not liking them a little (36 %) or a lot (35 %). However, a similar trend was not found for girls’ perceived satisfaction with the club coaches and managers.

When asked via survey about what they liked most about the club, the majority of girls reported playing fun games/activities followed by dancing and recommended offering more and a greater variety of fun games/activities followed by more sports (e.g., basketball, swimming, volleyball, soccer, running), and dance (e.g., Zumba) in a future program. Girls appreciated receiving a healthy snack both before and after the club but wanted greater variety and a larger quantity of food. Girls also indicated that the main reason stopping them from attending the club 3 days a week was that they had other commitments (e.g., responsibilities at home; involvement in sports; homework; other plans or important things to do). Girls were not specifically asked about what they did not like about the club, but only 0–1 % of the girls each year reported they did not like it.

Fifty-five club coaches/managers completed the 3-item satisfaction survey. Overall, 89 % (*n* = 49) perceived the girls liked the PAs they conducted in the club, and 98.2 % (*n* = 54) thought the girls liked them as a coach in the club, and 92.7 % (*n* = 51) perceived the design/structure of the club was age-appropriate. Coaches/managers’ perceptions did not change significantly over the 3 years. In addition, 47 (85.5 %) of the 55 club coaches and managers reported the training prepared them for their responsibilities in the club.

### Fidelity

As evidenced from the mean survey scores in Table 6, process evaluators perceived that the PA club was well-received by the girls and delivered with high quality by the coaches/managers. Process evaluators indicated that the coaches/managers performed better with regard to giving girls some choices, using positive praise, emphasizing the need to be physically active, and helping each girl feel connected to others in years 2 and 3, as compared to year 1. In addition, girls perceived the club was successful in increasing their PA. Girls reported that the coaches/managers improved in using positive praise, being good role models, emphasizing the need to be physically active, making the club fun, helping girls feel connected to others, and assisting them to increase their PA skills over the 3 intervention years. Perceptions among coaches/managers did not change over the 3 intervention years. Although some improvement is needed in a few areas, most of the Table 6 data indicate that the club reflected the constructs of the HPM and SDT.

**Table 6:** Fidelity: theoretical integrity - % (n) of selecting ‘agree a little’ or ‘agree a lot’

Item	Theoretical constructs	Year 1			Year 2			Year 3			Total		
		Process evaluators	Girls	Coaches managers	Process evaluators	Girls	Coaches managers	Process evaluators	Girls	Coaches managers	Process evaluators	Girls	Coaches managers
Gave girls some choice	SDT: autonomy	63.7 % (14)*	67.1 % (112)	91.7 % (11)	85.7 % (30)	73.3 % (96)	84.2 % (16)	94.4 % (34)	81.7 % (125)	87.5 % (21)	83.9 % (78)	73.8 % (333)	87.2 % (48)
Used positive praise to reinforce good performance/behavior	HPM: self-efficacy & social support SDT: motivation	81.9 % (18)*	88 % (147)*	100 % (12)	94.3 % (33)	87.8 % (115)	100 % (19)	100 % (36)	92.2 % (141)	100 % (24)	93.5 % (87)	89.4 % (403)	100 % (55)
Served as good role model for PA	HPM: modeling	81.8 % (18)	68.9 % (115)*	83.3 % (10)	97.2 % (34)	84 % (110)	94.8 % (18)	91.6 % (33)	75 % (130)	83.4 % (20)	91.4 % (85)	78.7 % (355)	87.3 % (48)
Emphasized the need to be physically active outside the club.	HPM: norms	18.2 % (4)*	80.2 % (134)*	100 % (12)	74.3 % (26)	86.3 % (113)	94.8 % (18)	94.5 % (34)	90.2 % (138)	100 % (24)	68.9 % (64)	85.4 % (385)	98.2 (54)
Made the club fun for the girls.	HPM: enjoyment	100 % (22)	79.6 % (133)*	100 % (12)	100 % (35)	93.1 % (122)	100 % (19)	97.2 % (35)	94.8 % (271)	100 % (24)	98.9 % (92)	89.7 % (400)	100 % (55)
Motivated each girl to increase her MVPA in the PA club.	HPM: social support SDT: motivation	81.9 % (18)	83.8 % (140)*	100 % (12)	97.1 % (34)	86.3 % (113)	94.8 % (18)	97.2 % (35)	90.2 % (138)	100 % (24)	93.5 % (87)	86.7 % (391)	98.2 %(54)
Helped each girl feel connected to others in the club.	HPM: benefitsSDT: relatedness	77.3 % (17)*	81.9 % (120)*	91.6 % (11)	100 % (35)	83.2 % (109)	100 % (19)	97.2 % (35)	86.9 % (133)	100 % (24)	93.5 % (87)	80.3 % (362)	98.2 % (54)
Helped each girl increase her skills for doing physical activity or sports.	HPM: benefits, self-efficacy, & barriers SDT: competence	81.9 % (18)	83.2 % (139)*	58.4 % (7)	91.4 % (32)	85.5 % (112)	68.4 % (13)	97.2 % (35)	85 % (230)	91.7 % (22)	91.4 % (85)	86.9 % (392)	76.4 % (42)
Helped girls increase their PA	HPM: social support	__	86.8 % (145)	75 % (9)	__	89.3 % (117)	94.8 % (18)	__	88.9 % (136)	91.7 % (22)	__	88.2 % (398)	89.1 % (49)
Helped girls see a lots of reasons for doing PA	HPM: benefits	__	80.2 % (134)*	100 % (12)	__	86.3 % (113)	94.8 % (18)	__	90.2 % (138)	100 % (24)	__	85.4 % (385)	98.2 % (54)
Helped girls solve problems that stop them from being active	HPM: barriers	__	71.3 % (119)	100 % (12)	__	82.4 % (108)	94.8 % (18)	__	78.4 % (120)	95.8 % (23)	__	76.9 % (616)	96.4 % (53)

*Note*. Survey items related to the theoretical constructs differed slightly for each of the 3 groups. The specific survey items that each group responded to are depicted in Table 2

*Significant at *p* < .05 when comparing to years 2 and 3

## Discussion

The process evaluation provided important information on the reach, dose, and fidelity related to an after-school PA club. The club was a component of a comprehensive intervention in a group randomized controlled trial to help adolescent girls, who are predominately African-American and of low SES, increase their MVPA. According to Griffin and colleagues [51], few trials involving an intervention to increase childhood and adolescent PA have undertaken comprehensive process evaluations. A detailed process evaluation is essential to illuminate what is causing or hindering expected changes resulting from an intervention [52]. This study was designed to contribute toward addressing this gap in information.

Although reach was lower than anticipated, which certainly may have influenced the study outcomes, the average attendance of 41 % did fall within the range of 40–78 % reported for an after-school PA program offered 3 days/week for boys and girls of an age similar to the girls in this study [42]. Unfortunately, average attendance was not presented separately for the boys and girls. In a study involving only 11- to 12-year-old girls that included an after-school dance program 2 days a week for 20 weeks, only about 1/3 (*n* = 93) of the 284 enrolled girls attended 2/3 (*n* = 26–27) of the sessions [13, 53]. Even when a younger age group of 8- to 10-year-old African American girls was included in another study involving after–school dance, maintaining attendance was a problem with 107 girls (80 %) attending an average of ≤ 1 day a week of the 5 days per week that the program was offered [54]. Consistent with findings from this study, Jago and colleagues [40] found that a major barrier to attendance by both boys and girls in an after-school PA program offered 2 days a week for 20 weeks was having prior commitments. Although after-school programs are recommended for increasing PA [15], Jago et al. suggested that the attendance commitment may need to be reduced by offering them only once per week for fewer than 20 weeks [13].

Although trying to meet the needs of girls so that they are able and want to attend the club is important, other factors have to be considered as well, including the cost and resources (e.g., space requested in the school and staff hired) needed to operate it when only a small number of girls may attend each day, especially if transportation home by school bus is needed. Another issue is that some evidence [40] shows that clubs may be difficult to lead when attendance is low (e.g., not enough present to play a sport or fun game). Offering the club one day a week may prevent some girls from participating, but an adequate number may be able to attend regularly on the selected day. Informing girls in advance (e.g., at the time of recruitment) that if their school is randomly selected to receive the intervention, the club will run on a certain day for a specific number of weeks; and if they do not foresee that they can attend every week on this day for the specified number of weeks, then they should not volunteer to participate. Sharing this information early on with girls may assist them with future planning, possibly resulting in increased attendance.

The intervention coordinator consistently provided feedback on the latest process evaluation results to the coaches during each of the 3 intervention years with no improvement in attendance in the first 2 years. As a result, in year 3, the following 2 major changes were included: 1) alteration in the way PAs were conducted and 2) the use of monetary incentives. Of note is that attendance in year 3 was significantly higher than in prior years. Although a definitive explanation for the significantly higher attendance in year 3, as compared to prior years, cannot be provided, girls’ heightened interest in monitoring their points for accuracy and obtaining the monetary incentives was particularly evident.

Although research shows that external rewards may undermine or diminish intrinsic motivation [55], SDT indicates that intrinsic motivation is catalyzed when individuals are in a condition that is conducive toward its expression [56]. Cognitive evaluation theory (CET), which is considered to be a subset of SDT, indicates that events, such as rewards, that conduce toward feelings of competence and autonomy during activity can enhance intrinsic motivation because both basic psychological needs are satisfied [57]. However, based on CET, intrinsic motivation only increases for activities that are inherently interesting to an individual. Unfortunately, many activities that children and adolescents have to perform in schools are not inherently interesting or enjoyable, and intrinsic motivation becomes weaker as they advance across academic grades. Therefore, rewards, if not perceived as too controlling, might originally help them get exposed to PA and this exposure might allow them to experience the activity’s intrinsically interesting properties resulting in a shift in orientation [56].

Both the mean minutes for MVPA offered by the coaches/managers (opportunity observed by process evaluators) and accelerometer-measured minutes of MVPA indicated that the aim of at least 30 min was not achieved in years 1, 2, or 3. As can be determined from the data presented in Table 5, the limited observed opportunity for MVPA may have resulted from the high number of minutes of management time that occurred, particularly in years 1 and 3, despite repeated attempts by the intervention coordinator to reduce it. Year 2 had the lowest management time and the highest number of minutes of observed opportunity for MVPA offered by the managers/coaches. Even so, year 2 resulted in the lowest number of minutes of accelerometer-measured MVPA. One plausible explanation is that year 2 included the oldest cohort of girls with none in the 5^th^ grade. Although reasons underlying why older girls were less active during the PAs than younger girls are not yet completely understood [5], research shows that perceived barriers to PA, such as hating to sweat and lacking motivation, increase as girls advance in age across adolescence [58, 59], possibly resulting in the lower intensity of PA exhibited during the program by older girls, as compared to younger ones.

In contrast in year 3, girls’ minutes of accelerometer-measured MVPA exceeded the number associated with the observed opportunity for MVPA offered by the coaches/managers. Although no definitive explanation for this unexpected finding can be given, the possibility exists that some girls’ may have engaged in MVPA during the club management time (not coded by process evaluators as an opportunity for MVPA) if some girls were moving around and not paying attention to the coaches. Dealing with disruptive situations may have resulted in the need for more management time than anticipated. Also probable is that girls may have simply increased the intensity of their PA on their own during unstructured time in year 3 in order to ensure receipt of points for active participation in PA during the club leading to the monetary incentive. In a 2006 study by Vu and colleagues that involved focus groups, adolescent girls reported that monetary incentives can motivate girls to be physically active [60]. Regardless, of importance is that only girls who indicated they were not participating in or planning to be involved in PAs that involved MVPA and required participation 3 or more days per week after school were included in the study. Getting these relatively low-active girls moving during the PA club was a difficult task for the coaches/managers, possibly resulting in more management time and other non-MVPA time than anticipated.

Increasing MVPA in after-school programs has been identified as an arduous task. Even in after-school programs described as having a high level of intervention implementation adherence, only 29.3 % of participating girls achieved the anticipated 30 min of MVPA per day [52]. Enhancing PA promotion requires the manager and all coaches to work collaboratively to create a physical-activity-friendly environment by using strategies, such as encouraging girls to be active and engaging in PA with them throughout the time of the program [52]. Despite repeated efforts by the intervention coordinator to encourage the coaches to actively participate in the PAs with the girls, the researchers noted when visiting the clubs that this situation was not always occurring on a consistent basis. As evidenced in areas of low SES, emotional exhaustion and burnout may result in limited energy for implementing a new program [61–63], particularly one that is offered 3 days a week. Although all school administrators agreed, prior to this study’s intervention, that adequate space would be available 3 days a week, some managers and coaches reported that other after-school activities periodically reduced the available space and girls did not want to go outside when temperatures were close to or below freezing. Competition with other school-based programs for participant time [30] and lack of adequate space for MVPA and cold weather [64] have been identified as potential barriers to successful implementation of interventions. This information lends additional support for Jago and colleagues’ [13] suggestion that offering an after-school program 1 day a week may be most feasible for the school, participants, and the staff conducting it.

Although the MVPA was lower than what the researchers had planned, the fidelity of intervention delivery was high. However, based on responses to some items from process evaluators, girls, and coaches/managers, coaches/managers may have overestimated their ability in certain areas to adhere to the theoretical integrity of the intervention. Unfortunately, comparing the findings of this study with those of others is difficult because detailed assessments of implementation fidelity are lacking; and, if reported, the definitions used and methods employed are inconsistent across studies [65].

The study had several strengths. One was that independent process evaluators, who were external to the study and not serving as interventionists or data collectors, evaluated the club. In addition to observation of the club by the process evaluators to report the PA offered, accelerometers were used to provide an objective measure of the girls’ PA participation during the club time. Evaluative data were provided not only by the process evaluators but also coaches, managers, and girls. A rigorously conducted process evaluation resulted from the comprehensive planning that occurred in advance.

Some limitations were evident. On the one hand, generalizability of the findings might be limited due to the inclusion of only urban schools in underserved areas in the Midwestern U.S.; but, on the other hand, involving girls of minority or low SES as participants might be viewed positively. Program implementers could not be completely blinded to the group assignment because they were delivering a program to promote PA among the girls. The intervention coordinator was aware of the study’s specific aims; however, to potentially decrease any risk of bias, specific aims were not shared with any coaches or managers. Relying on self-reported information via a survey lends itself to reliability issues and memory bias [66]. To overcome this problem, coaches, managers, and girls completed the surveys immediately after the intervention had ended [30]. Whether schools having a high level of active participation in PAs with girls and encouragement from coaches actually had better outcomes than other schools and whether coaches changed their behavior due to being observed by process evaluators was difficult to determine without video-taping every manager and coach during each club session at all schools. Also unclear is whether girls changed their PA due to being observed. Although the latter possibility certainly exists, it is unlikely that girls of this age, even if they changed their behavior initially as a result of being observed, would be concerned about sustaining it for an hour. Unfortunately, a time-intensive video-taping procedure extended beyond the scope of this large-scale study and was simply not feasible. Whether the coaches’ professional background had any effect on any outcomes is also difficult to evaluate for 2 reasons: 1) the education and expertise of the coaches in each school varied, and girls had contact with more than 1 coach at each session, and 2) a myriad of factors interacted to influence the quality of club delivery. For example, whether a coach’s professional background mattered more than either personality (being fun and personable) or some schools having more resources than others (e.g., swimming pools and/or vast amount of space or physical education equipment that girls could use to be physically active) is unknown.

## Conclusion

The process evaluation yielded relevant findings regarding the reach, dose and fidelity related to the implementation of an after-school PA club for girls. Important lessons learned and areas requiring attention in future investigations were elucidated. Research is needed to test approaches for enhancing attendance because inadequate reach may negatively impact study outcomes. Innovative strategies are also needed to increase girls’ MVPA in an after-school program. Specific, possibly multiple, reasons why some girls do better overall in a PA club (e.g., simply prefer to be physically active) than others are difficult to ascertain, but warrant investigation. To keep girls engaged and reduce behavioral problems, a variety of activities that appeal to girls needs to be offered. The effect of varied incentives and frequencies of distribution on the outcomes of interest warrant investigation.

## Abbreviations

ALT-PE, Academic Learning Time - Physical Education; BMI, body mass index; C, control; HPM, health promotion model; I, intervention; MVPA, moderate-to-vigorous physical activity; PA, physical activity; PI, principal investigator; SDT, self-determination theory; SES, socioeconomic status; SOFIT, system for observing fitness instruction time; SPSS, statistical package for the social sciences
